# Older men display elevated levels of senescence‐associated exercise‐responsive CD28^null^ angiogenic T cells compared with younger men

**DOI:** 10.14814/phy2.13697

**Published:** 2018-06-25

**Authors:** Mark Ross, Lesley Ingram, Guy Taylor, Eva Malone, Richard J. Simpson, Dan West, Geraint Florida‐James

**Affiliations:** ^1^ School of Applied Sciences Edinburgh Napier University Edinburgh United Kingdom; ^2^ Institute of Cellular Medicine Newcastle University Newcastle United Kingdom; ^3^ Department of Nutritional Sciences Department of Pediatrics Department of Immunobiology The University of Arizona Tucson Arizona

**Keywords:** Age, Angiogenic T cells, CD28, Exercise

## Abstract

Aging is associated with elevated cardiovascular disease risk. As a result of aging, endothelial dysfunction develops, partly due to a reduction in vascular regenerative ability. CD31^+^ T cells (angiogenic T cells; T_ANG_) possess highly angiogenic capabilities; however, these cells are significantly reduced in older populations. In addition, older populations possess significantly higher senescent and highly differentiated T‐cell levels in circulation, and these are reported to be highly exercise responsive. We investigated whether older adults display greater levels of circulating senescent (CD28^null^) T_ANG_ cells and whether these cells were more exercise responsive than CD28^+^ T_ANG_ cells. Young (18–25 years; *n* = 9) and older (60–75 years; *n* = 10) healthy men undertook a 30‐min cycling bout at 70% $\dot{V}$O_2_peak, with circulating T_ANG_ cells (CD3^+^ CD31^+^ CD28^+/null^; including CD4^+^ and CD8^+^ subsets) measured preexercise, postexercise, and 1 h post exercise by flow cytometry. Older adults displayed reduced basal levels of T_ANG_ cells (mean ± SEM: 410 ± 81 vs. 784 ± 118 cells·*μ*L, *P* = 0.017), despite a greater proportion of these cells being CD28^null^ (26.26 ± 5.08 vs. 13.36 ± 2.62%, *P* = 0.044). Exercise significantly increased the circulating number of T_ANG_ cells in both young and older men. However, in older men alone, exercise preferentially mobilized CD28^null^ CD8^+^ T_ANG_ cells compared with CD28^+^ T_ANG_ cells (time × phenotype interaction: *P* = 0.022; Δ74 ± 29 vs. Δ27 ± 15 cells·*μ*L, *P* = 0.059), with no such difference observed between these phenotypes in the young population. In conclusion, this is the first study to demonstrate that despite observing lower circulating numbers of T_ANG_ cells, older adults display greater levels of senescent T_ANG_ cells in comparison with younger individuals, and these cells are more exercise responsive than CD28^+^ T_ANG_ cells. Lower number of circulating T_ANG_ and greater levels of senescent‐associated CD28^null^ T_ANG_ may contribute to greater CVD risk with advancing age.

## Introduction

Cardiovascular disease (CVD) is the leading cause of death of humans in western civilization (Lozano et al. 2012), and one of the significant risk factors is advancing age. The aging‐associated increase in CVD risk is due to reductions in endothelial function (Black et al. 2009), stiffening of the arterial tree (Wen et al. 2015), as well as reduced angiogenic capabilities and endothelial regenerative abilities (Xia et al. 2012). Circulating cells play a significant role in maintaining endothelial homeostasis. Such cells include endothelial progenitor cells (EPCs), which have been found to be reduced in number in the circulation of older individuals (Thijssen et al. 2006; Ross et al. 2018), and reduced circulating number of EPCs in older individuals is highly predictive of mortality (Lu et al. 2016; Samman Tahhan et al. 2017).

In 2007, another subset of circulating angiogenic cells was discovered. These were a subset of T lymphocytes that express CD31 that were found to stimulate optimal differentiation and growth of EPCs in vitro (Hur et al. 2007). Further studies found that these cells were also reduced in the circulation of those with vascular diseases compared with healthy age‐matched controls (Rouhl et al. 2012). Recently, we have been able to demonstrate that older adults also display significantly lower levels of these angiogenic T cells (T_ANG_) than younger gender‐matched counterparts (Ross et al. 2018). These cells are thought to act in a different manner to EPCs, where they most likely promote endothelial regeneration and angiogenesis in a paracrine manner via the secretion of angiogenic factors VEGF and IL‐8 (Hur et al. 2007; Kushner et al. 2010a).

Exercise promotes endothelial function (Black et al. 2008), improves arterial compliance, and is known to stimulate angiogenesis (Delavar et al. 2014; Baum et al. 2015). A single bout of exercise is known to stimulates the movement of EPCs into the peripheral blood compartment in the postexercise period (van Craenenbroeck et al. 2008; Ross et al. 2014, 2018), which may offer a vasculogenic window of adaptation. Previous studies have also observed redistribution of T_ANG_ cells into the circulation with a single bout of exercise, in both young and older men (Ross et al. 2016, 2018). This T‐cell response is not uncommon, as many T‐cell subsets increase in circulation in response to acute exercise stimuli, such as naive T cells (T_NAÏVE_), cytotoxic T cells (T_CYTOTOXIC_), and regulatory T cells (T_REG_) (Brown et al. 2014; Spielmann et al. 2014; Ingram et al. 2015; Clifford et al. 2017). In addition to moving into the circulation in response to exercise, these T cells are thought to egress from the circulation within minutes of exercise cessation. However, older and younger individuals display different T‐cell responses to exercise, such as greater percentage change redistribution of T_CYTOTOXIC_ cells and senescent T cells (which lack the expression of the co‐stimulatory marker CD28), due to the age‐related expansion of these cells with time (Spielmann et al. 2014). CD28^null^ T cells are resistant to apoptosis (Vallejo et al. 2000), and the loss of CD28 expression has been related to the loss of immune system responsiveness in aging adults potentially due to diminished antigenic recognition (Bryl and Witkowski 2004). In a recent study, CD28^null^ T_ANG_ cells were significantly elevated in patients with cardiovascular risk factors and those with systemic lupus erythematosus (SLE) compared with healthy controls (Lopez et al. 2016). These cells were associated with increased circulating levels of TNF‐*α*, IL‐8, IFN‐*α*, and IL‐10, suggesting a role in promoting inflammatory conditions. In addition, these CD28^null^ T_ANG_ cells also co‐expressed other differentiation markers, including CD56, perforin, granzyme B, and IFN‐*γ*, as well as CD57 which support the theory that CD28^null^ T_ANG_ cells display a senescent profile (Lopez et al. 2016). CD28^+^ T_ANG_ cells did not express these markers; however, they did express CD27, a T‐cell differentiation marker (Hintzen et al. 1993), to a greater extent than CD28^null^ T_ANG_ cells.

Our aim was to investigate if older adults displayed greater proportion of T_ANG_ cells lacking CD28 expression, indicating a senescent T_ANG_ profile. In addition, due to the role of a single bout of exercise preferentially mobilizing highly differentiated T cells, we hypothesized that exercise would stimulate a preferential ingress of CD28^null^ T_ANG_ cells in both young and older adults, with a greater effect seen in older adults due to the expansion of CD28^null^ T cells in this age group.

## Materials and Methods

### Ethical approval

Ethical approval was granted by the Edinburgh Napier University Research and Ethics Governance Committee. Written informed consent was obtained from all participants prior to commencement of the study.

### Participants

This study is an extension of an already published study (Ross et al. 2018), and thus some participants have taken part in both studies. Nineteen healthy adult males (young: age 18–25 years, *n* = 9, older: age 60–75 years, *n* = 10) volunteered to take part in the study. Participants were physically active (take part in moderate‐intensity physical activity >2 times per week), nonobese (BMI<30), and nonsmokers and not taking any medication. In addition, participants were not suffering or had suffered from CVD or diabetes as determined by AHA/ACSM prescreening questionnaire (Balady et al. 1998). Participants were advised not to partake in any strenuous exercise for 72 h prior to the visits to the Human Performance Laboratory.

On arrival at the Human Performance Laboratory, participants were measured for height, body mass, and resting blood pressure (BP). BP was measured after a 5‐min supine rest using an automated BP machine and upper arm cuff (Nonin Puresat Avant 2120, Nonin Medical Inc, Minnesota, USA; Ultra‐Check^®^ Blood Pressure Cuff, Statcorp Medical, Florida, USA). Participant characteristics are shown in Table 1.

**Table 1:** Participant characteristics and exercise trial data

	Young (*n* = 9, 18–25 years)	Older (*n* = 10, 60–75 years)	*P*‐Value
Age (years)	23 ± 2	65 ± 3	<0.001***
Body mass index (BMI, kg·m^2^)	25.54 ± 3.50	26.08 ± 3.58	0.775
Systolic blood pressure (mmHg)	126 ± 13	124 ± 13	0.771
Diastolic blood pressure (mmHg)	65 ± 8	74 ± 6	0.012*
$\dot{V}$O_2_peak (L·min^−1^)	4.15 ± 0.42	2.79 ± 0.45	<0.001***
Power output @ $\dot{V}$O_2_peak (W)	325 ± 38	219 ± 27	<0.001***
Power output @ 70% $\dot{V}$O_2_peak(W)	230 ± 27	156 ± 23	<0.0001***
Heart rate @ 70% $\dot{V}$O_2_peak(% Maximum heart rate)	88 ± 5	85 ± 7	0.398

Values shown are mean ± standard deviation. *P < 0.05, ***P < 0.001 difference between age groups.

### Assessment of peak oxygen consumption

Individual's peak oxygen consumption ($\dot{V}$O_2_peak) was quantified as described previously (Ross et al. 2018). Briefly, participants undertook an incremental graded exercise test to exhaustion on a cycle ergometer (Lode Corival, Lode, the Netherlands) using online breath‐by‐breath gas analysis (Metalyzer 3B, Cortex, Germany). Heart rate (HR) was recorded throughout the maximal graded exercise test by HR telemetry (Polar, Finland), and ratings of perceived exertion (RPE) were also monitored throughout the test (Borg 1998). The exercise test was completed when participants reached volitional exhaustion.

### Acute exercise bout

Within 7 days on completion of the $\dot{V}$O_2_peak test, participants returned to the Human Performance Laboratory after an overnight fast to undertake a 30‐min cycling ergometer exercise bout at 70% of their predetermined $\dot{V}$O_2_peak. HR and RPE were monitored throughout the exercise trial. Participants were allowed to drink water *ab libitum*. All participants undertook the exercise bout at the same time of day.

### Blood sampling and CD31^+^ T‐cell phenotyping

Blood was taken from participants before, immediately postexercise and 1 h post exercise bout by a trained phlebotomist using venepuncture at each timepoint. Peripheral blood was drawn into 1 × 6 mL vacutainers spray‐coated with EDTA anti‐coagulant, and 1 × 6 mL serum gel vacutainer (BD Biosciences, UK), with the first 3 mL of peripheral blood discarded. Total blood differential leukocyte counts were determined using an automated hematology analyzer (XS 1000i, Sysmex, UK). Peripheral blood mononuclear cells (PBMC) were isolated using density gradient centrifugation as described elsewhere (Ross et al. 2016). PBMCs were stained with anti‐CD3‐FITC, anti‐CD31‐APC, and anti‐CD28‐PE‐Cy5 antibodies (all BD Biosciences, UK), with subanalysis of T‐cell subsets using anti‐CD4‐PE (Miltenyi‐Biotec, Germany) and anti‐CD8‐PE (BD Biosciences, UK) antibodies to determine CD4^+^ and CD8^+^ T_ANG_ cells. PBMCs incubated for 30 min prior to enumeration by flow cytometry.

Peripheral blood collected in the serum gel vacutainer was allowed to clot for 30 min prior to centrifugation (1500*g* for 10 min at 21°C). Serum was obtained and stored at −80°C for analysis of cytomegalovirus (CMV) serostatus.

### Analysis of cytomegalovirus seropositivity

Resting serum samples were analyzed for CMV serostatus using enzyme‐linked immunosorbent assay (ELISA) (BioCheck Inc, USA). Known standards were used to determine fluorescent cutoffs for determination of CMV serostatus (seropositive or seronegative). None of the individuals within the study reported as CMV seropositive.

### Flow cytometry

**Figure 1:**
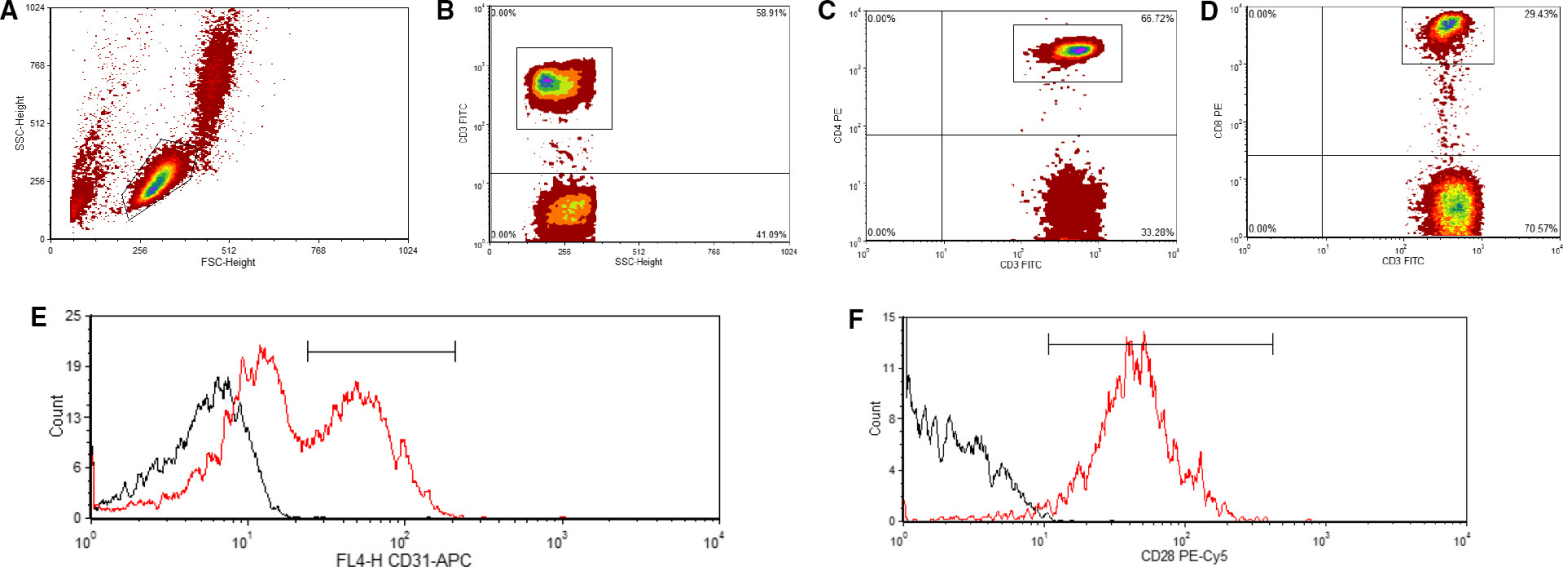
Flow cytometric quantification of CD31^+^ CD28^+/null^ T_ANG_ cells. Side scatter vs. forward scatter for identification of lymphocyte gate (A), CD3^+^ gating for identification of T cells (B), identification of CD4^+^ (C) or CD8^+^ (D) T cells followed by identification of CD31^+^ and CD31^−^subsets (E). CD31^+^ subsets were then analyzed for expression of CD28 (F). Histogram data shows isotype control (black lines) and sample (red lines).

Circulating cell data were obtained using CELLQuest Pro software (BD Biosciences, USA) on a BD FACS Calibur four‐color flow cytometer equipped with a 15 mW argon ion laser emitting light at fixed wavelength of 488 nm (BD Biosciences, USA). First, lymphocyte population was gated using forward scatter and side scatter. CD3^+^ events were gated, followed by gating of CD4^+^ and CD8^+^ populations. Subsequent expression of CD31 was gated for, and these cells were assessed for expression of CD28. Representative flow cytometry dot plots is provided in Figure 1; 10,000 lymphocytic events were measured per sample. Circulating concentrations of T cells and subsequent subsets were obtained using a dual platform method, by multiplying the percentage values obtained from the flow cytometer by the corresponding lymphocyte counts as obtained from hematology analysis.

Changes in blood volume were accounted for by using known measures of hematocrit and hemoglobin obtained from automated hematology analysis (Sysmex, XS 1000i, UK) (Dill and Costill 1974).

### Statistical analysis

All data are presented as mean ± SEM unless otherwise stated. Independent *t*‐tests were performed to identify differences in physical characteristics and exercise‐based measures between the young and older participants, as well as resting lymphocyte subsets between these two age groups. Linear mixed models were used to determine the main effects of exercise on cell numbers and interaction with age. When significant differences were detected, Bonferroni post hoc tests were performed to determine location of the effect (preexercise, postexercise, and 1 h post exercise). Where significant age group interactions were observed, Eta squared (ƞ^2^) values were calculated for effect size. Independent *t*‐tests were performed to compare the ingress (preexercise to immediately postexercise) and egress (immediately postexercise to 1 h post exercise) (presented as Δ in cells) of cell populations between age groups. Data were analyzed using SPSS for Macintosh, version 20 (IBM, Chicago, USA). Significance alpha was set at *P *<* *0.05.

## Results

### Resting circulating T‐cell subsets and age

**Figure 2:**
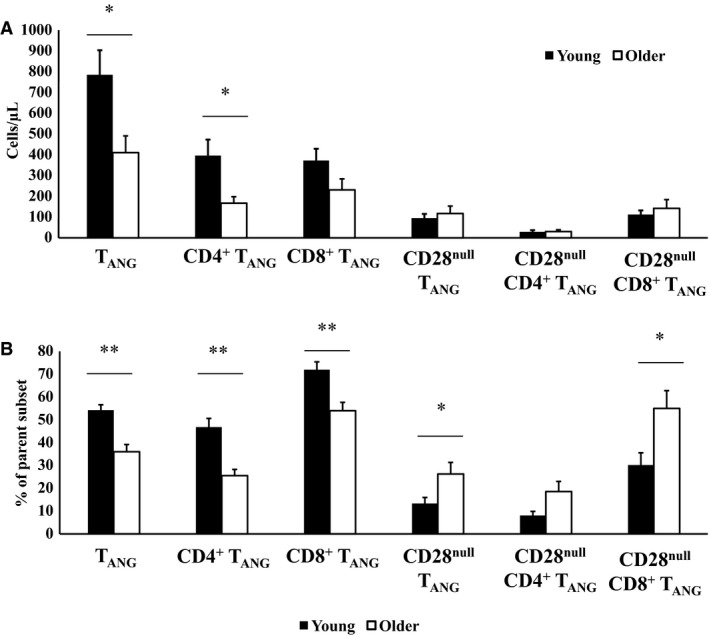
Basal levels of circulating total and CD28^null^ CD31^+^ T cells (T_ANG_) in healthy young (*n* = 9) and older (*n* = 10) men. (A) Absolute circulating numbers of T_ANG_ cells, (B) proportional levels of T_ANG_ cells and subsets. *Significant difference between young and older men (*P* < 0.05).

Older individuals displayed reduced absolute number of CD3^+^CD31^+^ T cells (T_ANG_ : 410 ± 81 vs. 784 ± 118 cells·*μ*L, *t*_*(1,18)*_ = 2.675, *P* = 0.017) and CD4^+^ T_ANG_ cells (167 ± 31 vs. 396 ± 77 cells·*μ*L, *t*_*(1,18)*_ = 2.883, *P* = 0.021), with a trend toward reduced number of CD8^+^ T_ANG_ cells (231 ± 53 vs. 372 ± 57 cells·*μ*L, *t*_*(1,18)*_ = 1.802, *P* = 0.092) compared with the younger cohort. This was reflected with a significantly reduced proportion of T cells expressing CD31 (CD3^+ ^CD31^+^: 36.00 ± 3.19 vs. 54.24 ± 2.37%, *t*_*(1,18)*_ = 4.491, *P* = 0.000; CD3^+ ^CD4^+ ^CD31^+^: 25.52 ± 2.74 vs. 46.82 ± 3.82%, *t*_*(1,18)*_ = 4.604, *P* = 0.000; CD3^+ ^CD8^+ ^CD31^+^: 54.00 ± 3.68 vs. 71.97 ± 3.45%, *t*_*(1,18)*_ = 3.537, *P* = 0.003) in older men compared with the younger men (Fig. 2A). These differences were not a result of any differences in total CD3^+^, CD4^+^, or CD8^+^ T cells between the age groups (*P* > 0.05, data not shown).

Older men also displayed increased percentage of CD28^null^ T_ANG_ cells (26.26 ± 5.08 vs. 13.36 ± 2.62%, *t*_*(1,18)*_ = 2.260, *P* = 0.044) and CD28^null^ CD8^+^ T_ANG_ cells (55.00 ± 7.81 vs. 30.19 ± 5.32%, *t*_*(1,18)*_ = 2.189, *P* = 0.021), with a trend for increased levels of CD28^null^ CD4^+^ T_ANG_ cells (18.55 ± 4.44 vs. 8.07 ± 1.81%, *t*_*(1,18)*_ = 2.466, *P* = 0.052) compared with younger men (Fig. 2B).

### Influence of age on redistribution of T_ANG_ cells

**Figure 3:**
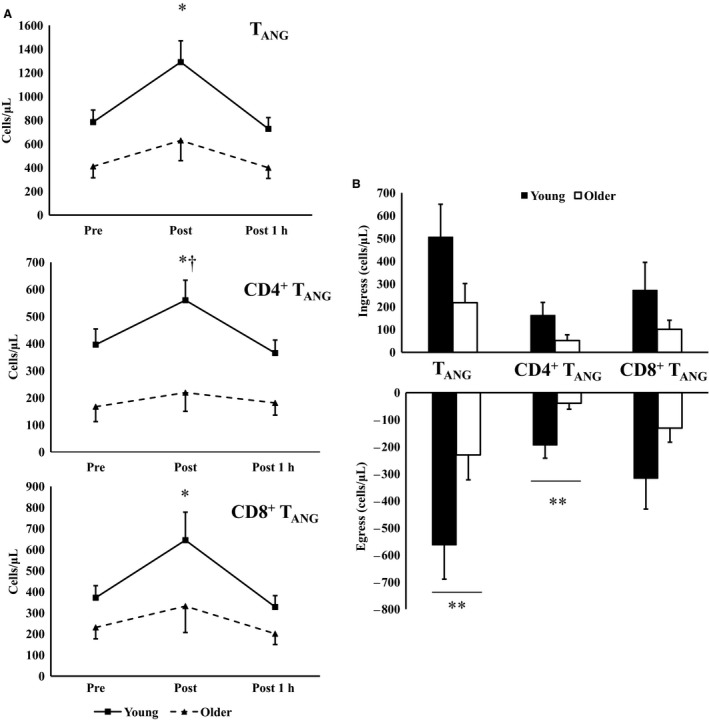
CD31^+^ T‐cell response to acute exercise bout in healthy young (*n* = 9) and older men (*n* = 10). (A) Absolute cell changes in response to exercise, (B) ingress and egress of T_ANG_ cell subsets to exercise in young and older men. *Significant main effect of exercise, †Significant exercise × age interaction effects (*P* < 0.05). B – **significant difference between young and older individuals (*P* < 0.05).

The effects of age on the exercise responsiveness of T_ANG_ cells and subsets are shown in Table 2. The number of T_ANG_ (*F*_*(2,18)*_ = 22.017, *P* = 0.001), CD4^+^ T_ANG_ (*F*_*(2,18)*_ = 13.718, *P* = 0.002), and CD8^+^ T_ANG_ (*F*_*(2,18)*_ = 11.583, *P* = 0.002) cell subsets increased immediately after the acute exercise bout compared with baseline and 1 h after exercise. There was a significant time × age interaction in the response of CD4^+^ T_ANG_ cells to the exercise bout (*F*_*(2,18)*_ = 5.250, *P *=* *0.011). No time × age interaction was found for total T_ANG_ or CD8^+^ T_ANG_ cells. The exercise responsiveness of T_ANG_ cells based on age is described in Figure 3A.

**Table 2:** Exercise‐induced changes in T_ANG_ cells and subsets in healthy males (18–25 years *n* = 9, 60–75 years, *n* = 10) contrasted by age

Cell subset	Pre	Post (cells·μL^−1^)	1 h Post	Main effects of time	Main effects of age	Interaction: time × age
Total T_ANG_		#†				
18–25 years	784 ± 102	1290 ± 180	727 ± 95	*F*_*(2,18)*_ = 22.107; *P *=* *0.000*, *ƞ* ^2^ = 0.595	*F*_*(2,18)*_ * *= 7.719; *P*=* *0.014*, *ƞ* ^2^ = 0.34	*F*_*(2,18)*_ = 3.731; *P*=* *0.036*, *ƞ* ^2^ = 0.20
60–75 years	410 ± 727	629 ± 399	399 ± 90			
CD4^+^ T_ANG_		#†				
18–25 years	396 ± 58	560 ± 74	365 ± 48	*F*_*(2,18)*_ = 13.718; *P *=* *0.000*, *ƞ* ^2^ = 0.48	*F*_*(2,18)*_ = 10.313; *P *=* *0.006*, *ƞ* ^2^ = 0.41	*F*_*(2,18)*_ = 5.250; *P *=* *0.011*, *ƞ* ^2^ = 0.26
60–75 years	167 ± 55	219 ± 69	181 ± 45			
CD8^+^ T_ANG_		#†				
18–25 years	372 ± 57	645 ± 133	328 ± 54	*F*_*(2,18)*_ = 11.583; *P *=* *0.000*, *ƞ* ^2^ = 0.44	*F*_*(2,18)*_ = 3.198; *P *=* *0.094, *ƞ* ^2^ = 0.18	*F*_*(2,18)*_ = 2.153; *P *=* *0.134, *ƞ* ^2^ = 0.13
60–75 years	231 ± 54	332 ± 125	201 ± 51			
CD28^null^ T_ANG_		#†				
18–25 years	95 ± 33	268 ± 113	91 ± 33	*F*_*(2,18)*_ = 6.384; *P *=* *0.005*, *ƞ* ^2^ = 0.31	*F*_*(2,18)*_ = 0.000; *P *=* *0.984, *ƞ* ^2^ = 0.00	*F*_*(2,18)*_ = 0.139; *P *=* *0.871, *ƞ* ^2^ = 0.01
60–75 years	117 ± 29	240 ± 99	102 ± 27			
CD28^null^ CD4^+^ T_ANG_		#†				
18–25 years	29 ± 10	58 ± 13	49 ± 12	*F*_*(2,18)*_ = 2.834; *P *=* *0.076, *ƞ* ^2^ = 0.17	*F*_*(2,18)*_ = 1.098; *P *=* *0.312, *ƞ* ^2^ = 0.07	*F*_*(2,18)*_ * *= 1.707; *P *=* *0.200, *ƞ* ^2^ = 0.11
60–75 years	30 ± 8	35 ± 12	30 ± 11			
CD28^null^ CD8^+^ T_ANG_		#†				
18–25 years	112 ± 36	241 ± 78	96 ± 32	*F*_*(2, 18)*_ * *= 9.762; *P *=* *0.001*, *ƞ* ^2^ = 0.39	*F*_*(2,18)*_ * *= 0.200; *P *=* *0.889, *ƞ* ^2^ = 0.00	*F*_*(2,18)*_ * *= 0.477; *P *=* *0.625, *ƞ* ^2^ = 0.03
60–75 years	142 ± 33	217 ± 74	117 ± 30			

Main effects and interaction effects of time and age are presented. # and † indicate significant differences from postexercise to preexercise and 1 h post exercise, respectively (*P* < 0.05). Significant main and interaction effects denoted by * (*P* < 0.05) effects. Data are presented as mean ± SEM.

The mobilization and egress of T_ANG_ cells were greater in younger individuals, although statistical significance was only observed for the egress of T_ANG_ cells (563 ± 126 vs. 230 ± 92 cells·*μ*L, *t*_*(1,18)*_ **= 2.174, *P *=* *0.046), with a trend for greater ingress of both total T_ANG_ cells (*P* = 0.098) and CD4^+^ T_ANG_ cells (*P* = 0.078) in young vs. older men. Similar patterns were observed for both CD4^+^ and CD8^+^ T_ANG_ cells, however, only statistical significantly greater egress for CD4^+^ T_ANG_ cells (194 ± 48 vs. 39 ± 22 cells·*μ*L, *t*_*(1,18)*_ **= 3.036, *P *=* *0.008) (Fig. 3B).

### Influence of age on CD28^+/null^ T_ANG_ cells in response to exercise

There was an increase in CD28^null^ T_ANG_ cells immediately postexercise compared with preexercise and 1 h post exercise regardless of age (total T_ANG_, *F*_*(2,18)*_ = 6.384, *P *=* *0.005; CD4^+^ T_ANG_, *F*_*(2,18)*_ = 2.834, *P *=* *0.076; CD8^+^ T_ANG_, *F*_*(2,18)*_ = 9.462, *P* = 0.001). In addition, there were no differences between the young and older group in the mobilization and subsequent egress of CD28^null^ T_ANG_ cells in response to exercise (*P* > 0.05; Table**** 2).

**Figure 4:**
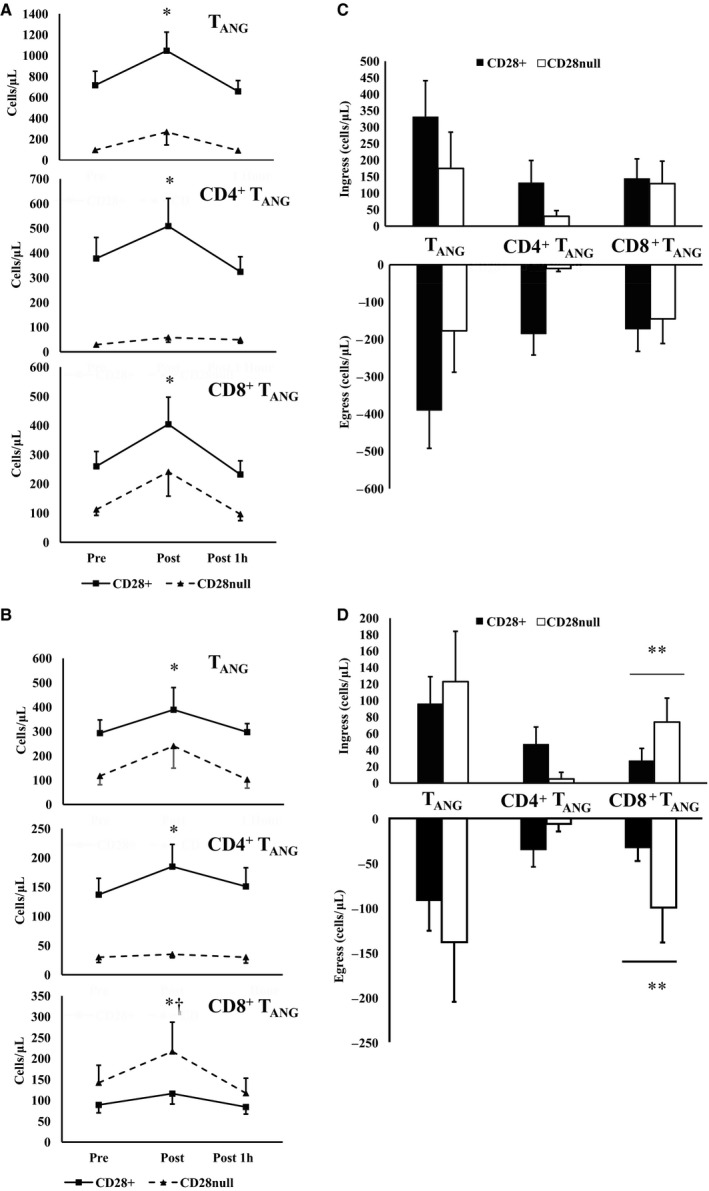
Exercise responsiveness of CD28^+^ and senescent‐associated CD28^null^ T_ANG_ cells in young (*n* = 9; A and C) and older (*n* = 10; B and D) men. *Significant main effect of exercise, † significant exercise × phenotype interaction effects (*P* < 0.05). D – **significant difference ingress and egress between CD28^null^ and CD28^+^ CD8^+^ T_ANG_ cells in older individuals (*P* < 0.05).

In the young cohort, there were no differences in the exercise responsiveness of CD28^null^ vs. CD28^+^ T_ANG_ cells and subsets (*P* < 0.05). This was also reflected in no statistical differences in absolute cell mobilization or egress. However, in the older group, there was a significant time × phenotype interaction for CD8^+^ T_ANG_ cells (*F*_*(2,18)*_ = 4.882, *P* = 0.022), with a greater ingress (74 ± 29 vs. 27 ± 15 cells·*μ*L, *t*_*(1,18)*_ **= 2.203, *P* = 0.059) and egress (99 ± 39 vs. 32 ± 15 cells·*μ*L, *t*_*(1,18)*_ = 2.375, *P* = 0.045) of CD28^null^ CD8^+^ T_ANG_ cells than CD28^+^ CD8^+^ T_ANG_ cells (Fig. 4).

## Discussion

This is the first study to investigate the influence of age and exercise on T_ANG_ cell redeployment, and specifically senescence‐associated CD28^null^ T_ANG_ cells. We report that older adults display reduced number of circulating T_ANG_ cells (including CD4^+^ and CD8^+^ subsets), but also display increased proportion of T_ANG_ cells lacking CD28 expression which is associated with a senescent T_ANG_ profile (Lopez et al. 2016). Our results also show that older adults display a blunted responsiveness of T_ANG_ cells to moderate intensity exercise. This effect included an apparent blunted ingress of these cells into the circulation during exercise and a blunted egress of cells from circulation 1 h post exercise. However, in contrast with our previous study, our ingress data did not reach statistical significance (*P* = 0.098 for trend), despite >Δ280 cells·*μ*L^−1^ difference between young and older men in our study (total T_ANG_ cells), which may be of clinical significance. Interestingly, we also show that in the young population (18–25 years) that there were no differences in the response of CD28^null^ and CD28^+^ T_ANG_ cells; however, in the older population (60–75 years), there was a greater responsiveness of CD28^null^ than CD28‐expressing CD8^+^ T_ANG_ cells.

Our lab has previously shown that exercise significantly increases the number of circulating T_ANG_ cells (Ross et al. 2016), and older adults display reduced resting and exercise‐induced mobilization of T_ANG_ cells into the circulation in response to an exercise bout (Ross et al. 2018). Reductions in basal T_ANG_ cells in older adults may be due to thymic involution (Simpson 2011); however, we do observe an increase in CD28^null^ T_ANG_ cells in the older population. CD28 expression is lost on repeated rounds of T‐cell division and/or encounters with antigens (Vallejo 2005), and CD28^null^ T cells are apoptotic resistant and linked with reduced immune efficacy (Bryl and Witkowski 2004). Recently, CD28^null^ T_ANG_ cells were shown to be reduced in individuals with elevated cardiovascular risk factors and in those with SLE than healthy age‐matched controls (Lopez et al. 2016). These cells were also characterized as expressing granzyme B, perforin, IFN‐*γ*, and markers, such as CD57, CCR7, and CD56, indicative of a senescent and cytotoxic profile. However, the study by Lopez et al. (2016), only quantified CD4^+^ T_ANG_ cells and subsequent expression of CD28, and so the current work are the first report of greater proportion of CD8^+^ T_ANG_ cells being CD28^null^ between different human populations. The increase in the senescent T_ANG_ cell population was recently associated with elevated plasma inflammatory cytokines, such as IFN‐*α*, IL‐10, TNF‐*α*, and IL‐8 (Lopez et al. 2016), suggestive of a pro‐inflammatory role, which may further exacerbate the increased CVD risk in older individuals who display greater levels of these senescent T cells.

Single bouts of exercise are well reported to stimulate redistribution of T cells into the circulation in humans (Simpson et al. 2007; Turner et al. 2010; Witard et al. 2012), with age differences apparent for various subpopulations of T cells (Spielmann et al. 2014; Bigley et al. 2015; Ross et al. 2018). In this study, we show that older adults mobilize T_ANG_ cells to a lesser extent than younger adults, albeit not statistically significant, with younger individuals mobilizing over 2× more T_ANG_ cells in response to the exercise stressor. Both CD4^+^ (~3.2x) and CD8^+^ (~×2.7) subsets show similar patterns. Younger adults also demonstrated a greater egress of cells away from the circulation 1 h post exercise, which may represent greater immune cell trafficking to potential sites of infection or tissue damage, and hence greater immune system efficiency. Kushner et al. (2010b) demonstrated greater T_ANG_ cell migration in vitro compared with T_ANG_ cells from older adults which may partially explain the greater egress observed in our study. The greater redeployment of these cells may simply be representative of greater numbers of these cells in younger populations rather than enhanced mobilization ability *per se*.

The T‐cell response to exercise is highly dependent on the phenotype studied. It has been well reported that highly differentiated, senescent, and cytotoxic T cells display greater exercise responsiveness than naive and low differentiated T‐cell subsets (Simpson et al. 2007, 2010; Spielmann et al. 2014) potentially due to greater β_2_‐adrenergic receptor expression (Fan and Wang 2009). Senescent and highly differentiated T cells typically lack CD28 expression (Vallejo 2005; Parish et al. 2010), and thus, these cells can be somewhat identified in human peripheral blood via staining with CD28 antibody. In agreement with other studies (Simpson et al. 2007, 2008), we have shown in this study that senescence‐associated CD28^null^ T_ANG_ cells are more exercise responsive than CD28^+^ counterparts, but only in older adults, and only in the CD8^+^ subset. The age‐related expansion of these senescent T cells (Spielmann et al. 2011), as well as these cells displaying greater sensitivity to exercise‐induced β_2_‐adrenergic stimulation, may explain this finding (Kruger et al. 2008; Anane et al. 2009). We did not observe this preferential mobilization in the younger cohort. Aging is also linked with decreased lymphocyte β_2_‐adrenergic sensitivity (O'Hara et al. 1985), together with the importance of β_2_‐adrenergic signaling for exercise‐induced T‐cell redeployment (Kruger et al. 2008), this may partly explain differences of T_ANG_ responses to exercise between young and older individuals. Other factors known to modulate the aging‐associated differences in T‐cell subset responses to exercise include infection history. Turner et al. (2010) and Spielmann et al. (2014) have both reported that those who are cytomegalovirus seropositive (CMV^+^) display a greater redistribution of senescent T cells than CMV seronegative individuals (CMV^−^) due to the expansion of these cells due to the latent virus over time. Considering this, we tested all participants for serostatus and none of the individuals in either young or older adult group were CMV^+^, suggesting our findings are due to age rather than differences in infection history.

T_ANG_ cells are reportedly highly vasculogenic (Hur et al. 2007; Kushner et al. 2010a) and are associated with endothelial function scores (Kushner et al. 2010a). We and others have observed a reduction in these cells in older individuals compared with younger sex‐matched cohorts (Kushner et al. 2010b; Ross et al. 2018), which may contribute to the increased CVD risk among the elderly. These cells can reportedly augment vasculogenesis of resident endothelial cells or endothelial progenitors via paracrine means. As yet, we do not know of any vasculogenic functional changes with age in this angiogenic cell population; however, we may surmise that a senescent and highly differentiated profile may coincide with losing angiogenic capabilities, as it is associated with loss of immunological function (Bryl and Witkowski 2004). However, this is yet to be confirmed and is an avenue for future study. We also propose that exercise may offer a short window of opportunity, whereby mobilization of T_ANG_ cells into the circulation exposes the endothelium to an increase in these cells, therefore allowing the T_ANG_ cells to act on this target tissue. In the older population, however, this window of opportunity appears to be blunted due to the observed trend for an impaired response of total T_ANG_ cell subsets and augmented ingress of senescent cells, which may have implications for angiogenic function of these cells.

## Conclusion

In summary, we have shown for the first time that although displaying reduced circulating T_ANG_ cells compared with younger adults, older individuals display greater levels of senescence among their circulating T_ANG_ cells, and these senescent T_ANG_ cells are preferentially mobilized in response to exercise compared with CD28^+^ T_ANG_ cells. These changes in T_ANG_ cell numbers and subsets may contribute to elevated CVD risk among an elderly population.

## Conflict of interest

The authors declare that they have no conflict of interest.
